# Effects of massage as a recuperative technique on autonomic modulation of heart rate and cardiorespiratory parameters: a study protocol for a randomized clinical trial

**DOI:** 10.1186/s13063-018-2830-1

**Published:** 2018-08-25

**Authors:** Nilton Mantovani Junior, Eduardo Pizzo Junior, Malu dos Santos Siqueira, Allysiê Priscilla de Souza Cavina, Carlos Marcelo Pastre, Franciele Marques Vanderlei

**Affiliations:** 1 Postgraduate Program in Physiotherapy, Universidade Estadual Paulista (FCT/UNESP), Presidente Prudente, São Paulo Brazil; 2 Graduation in Physiotherapy, Universidade Estadual Paulista (FCT/UNESP), Presidente Prudente, São Paulo Brazil; 3 Department of Physiotherapy, Universidade Estadual Paulista (FCT/UNESP), Presidente Prudente, São Paulo Brazil

**Keywords:** Autonomic nervous system, Massage, Recovery of function, Heart rate

## Abstract

**Background:**

Recuperative techniques have been used to anticipate and potentiate recovery. The massage is one of the most widely used in sports. Among the ways to demonstrate the recovery of the organism is the resumption of autonomic modulation of heart rate, which can be analyzed in situations that cause disturbances in the behavior of the cardiovascular system with the objective of verifying the responsiveness of the autonomic nervous system (ANS). Recovery can be assessed through heart rate variability (HRV) which analyzes the oscillations in consecutive heartbeats, thus allowing an important non-invasive alternative for the study of modulation of the ANS. The objective of the study will be to measure the effects of massage as a recuperative technique on the autonomic modulation of heart rate and cardiorespiratory parameters at different moments of application.

**Methods:**

This is a randomized, cross-over clinical trial. Forty men aged 18 to 30 years, healthy and physically active according to the International Physical Activity Questionnaire will participate in the study. Participants will be randomized into groups, which will perform the five interventions of the study at randomized moments, one intervention per session: Intervention 1: control; Intervention 2: participants will receive the massage protocol; Intervention 3: performance of the stress protocol; Intervention 4: participants will perform the stress protocol and immediately after receive the massage; Intervention 5: participants will perform the stress protocol and 1 h after conclusion of the protocol will receive the massage. The sessions will occur with an interval of 1 week between them and, due to the technique used, blinding participants and therapists is not possible. The primary outcome measure is HRV that will be measured 2 h after the conclusion of each intervention, and secondary outcome measures, which include heart rate, respiratory rate, blood pressure, oxygen saturation, and individual touch perception, will be measured at specific moments in the course of each intervention.

**Discussion:**

The implementation and use of this standardized protocol should provide important and reliable information regarding the use of massage in post-exercise recovery, with the identification of its effects on the ANS and the best timing and form of massage application. The data obtained in the present study will provide subsidies for the best management of application of the technique in sports clinical practice, considering periods of training and, mainly, of competitions.

**Trial registration:**

ClinicalTrials.gov, ID:NCT03094676. Pre-results. 12 March 2018.

**Electronic supplementary material:**

The online version of this article (10.1186/s13063-018-2830-1) contains supplementary material, which is available to authorized users.

## Background

Training in its simplest form represents acute challenges to the body intended to optimize chronic improvements in physiological capabilities [1]. The success of the processes of performance improvement and injury prevention depends on the quality of the transition between the physical training stimuli, in addition to the systematization of the exercise prescription. In this sense, an adequate recovery becomes an important aspect of any conditioning program, both for athletes, technicians, and for several health-related professionals [1].

Physical exercise can be a stressor mechanism to body systems, promoting several organic modifications, and recovery processes are commonly employed in the sense of restoring the systems to their basal condition [1]. To accelerate the recovery process after physical exercise, specific techniques are used, and early recovery is considered fundamental for performance, especially within the sporting scene [2]. Of these techniques, massage, consisting of the mechanical manipulation of the corporal tissues [2], stands out, being considered the most commonly used technique in the sports field, both in training and competition periods [3].

Clinical, functional, and metabolic modifications are already described in the relevant literature regarding the effectiveness of massage [4–9]. It is believed that the massage technique is beneficial in reducing muscle pain, by relieving muscle tension and edema as exposed by Hunter et al. [10], stimulating the metabolic clearance of blood lactate and creatine kinase as proposed by Smith et al. [11], and promoting improvement in flexibility and range of motion [6].

However, the physiological adaptations promoted by massage can also be monitored through autonomic nervous system (ANS) and cardiovascular responses [11]. Studies have shown that relaxing therapeutic modalities, such as massage, have presented favorable results for recovery in cardiovascular markers, such as a decrease in heart rate and blood pressure in the post-exercise period [12–14].

In relation to the markers of autonomic recovery, massage has also demonstrated positive results in the recovery capacity. Arroyo-Morales et al. [15] performed a 40-min massage on the whole body of the participants after a three-sprint Wingate exercise protocol of 30 s separated by a 3-min interval and observed that massage was favorable for the recovery of the heart rate variability (HRV) indices to baseline.

However, in the study of Arroyo-Morales et al. [15] some gaps can be identified in the literature. Before the massage technique, the participants performed 15 min of active and passive recovery, the massage technique had five different modalities of tissue manipulation applied in regions that were not protagonists during the stress, with prolonged duration and applied only at one moment. These details in the design of the study may influence the responses found in the recovery of autonomic modulation of heart rate, especially considering practical applications. However, the main gap addressed by the present study protocol is related to the moment of application of massage as a recuperative technique. To study the optimal timing of the technique seems interesting since various sports modalities participate in several competitions throughout the day and, thus, it is necessary to understand the best moment of application of the recuperative technique to guarantee the best performance of the athletes.

The recovery of autonomic modulation of heart rate has been considered an important global marker of athlete recovery. Ihsan et al. [16] reported that the return of HRV values is a good indicator for exercise dynamics to be applied in training or in competitions. In addition, although massage is widely used as a recuperative technique, there are gaps in the literature regarding the effectiveness of massage in the autonomic modulation of heart rate and cardiorespiratory parameters; and especially in relation to the best moment of application. It is unclear whether performance of the massage technique has a beneficial effect on HRV both to anticipate its recovery and potentiate the effects of recovery depending on the moment of its application.

The present study is a randomized clinical trial aimed at investigating the effects of massage as a recuperative technique on the recovery of autonomic modulation of heart rate and cardiorespiratory parameters in physically active young people comparing two distinct scenarios: (1) immediately after stress in order to investigate if the technique anticipates the recovery of the ANS, and (2) immediately after the recovery moment of the ANS in order to investigate whether the technique potentiates the recovery of the ANS.

## Methods

### Study design

**Fig. 1:**
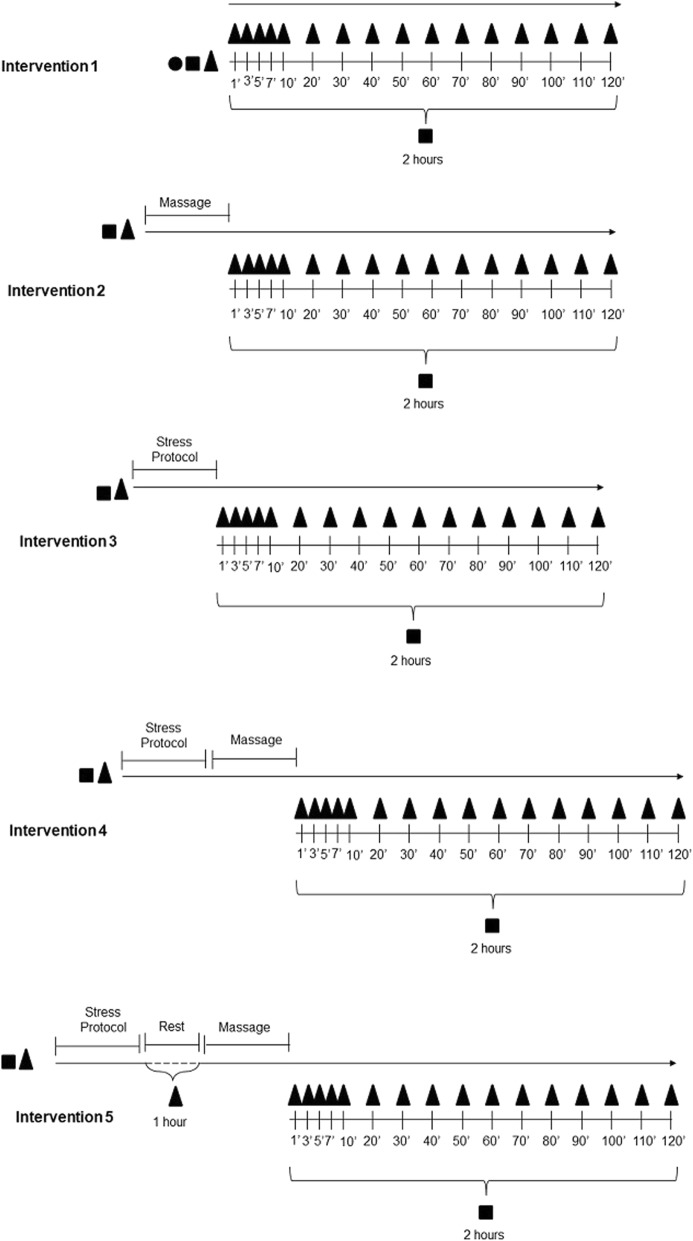
Study design. Legend: ● individual touch perception; ■ heart rate variability (HRV); ▲ cardiorespiratory parameters

A randomized, cross-over, controlled clinical trial will be conducted at the Center for Studies and Care in Physiotherapy and Rehabilitation of the Universidade Estadual Paulista (FCT/UNESP), Presidente Prudente, São Paulo, Brazil. The study design is illustrated in Fig. 1. The study protocol follows the SPIRIT 2013 Checklist (Standard Protocol Items: Recommendations for International Trials) (Additional file 1) and TIDieR (Template for Intervention Description and Replication) so that the information and quality of the reports of the interventions are well described. The trial was registered at ClinicalTrials.gov (NCT03094676).

### Participants

A total of 40 participants will be recruited at the local university (Universidade Estadual Paulista) through online advertising and approaching participants by telephone, SMS, and social networks, from January 2018 to April 2018. Participants will be considered eligible if they are apparently healthy, male, aged between 18 and 30 years, and have a Body Mass Index (BMI) classified as normal (between 18.5 and 25 kg.m^2^) according to the World Health Organization [17]. In addition, participants who have at least one of the following characteristics will not be included: smokers, use of medications that influence autonomic modulation of heart rate, alcoholics, known metabolic and/or endocrine disorders, individuals with musculotendinous injuries in the region of the lower limbs and trunk in the previous 6 months, and sedentary, insufficiently active, and very active individuals according to the International Physical Activity Questionnaire (IPAQ) [18]. With the exception of assessing the level of physical activity that will be carried out through the application of the IPAQ, the principal investigator will determine whether potential participants meet the eligibility criteria during an initial interview. Ethical approval has been granted by the Human Ethics Committee of the São Paulo State University (CAAE: 57584116.6.0000.5402).

### Analysis population

The criteria that define the exclusion of the participant in the final analysis of the data will be: intercurrences during the execution of the protocols that prevent their continuity, absence from more than 80% of the proposed sessions, and capture errors in the RR intervals.

### Randomization

**Fig. 2:**
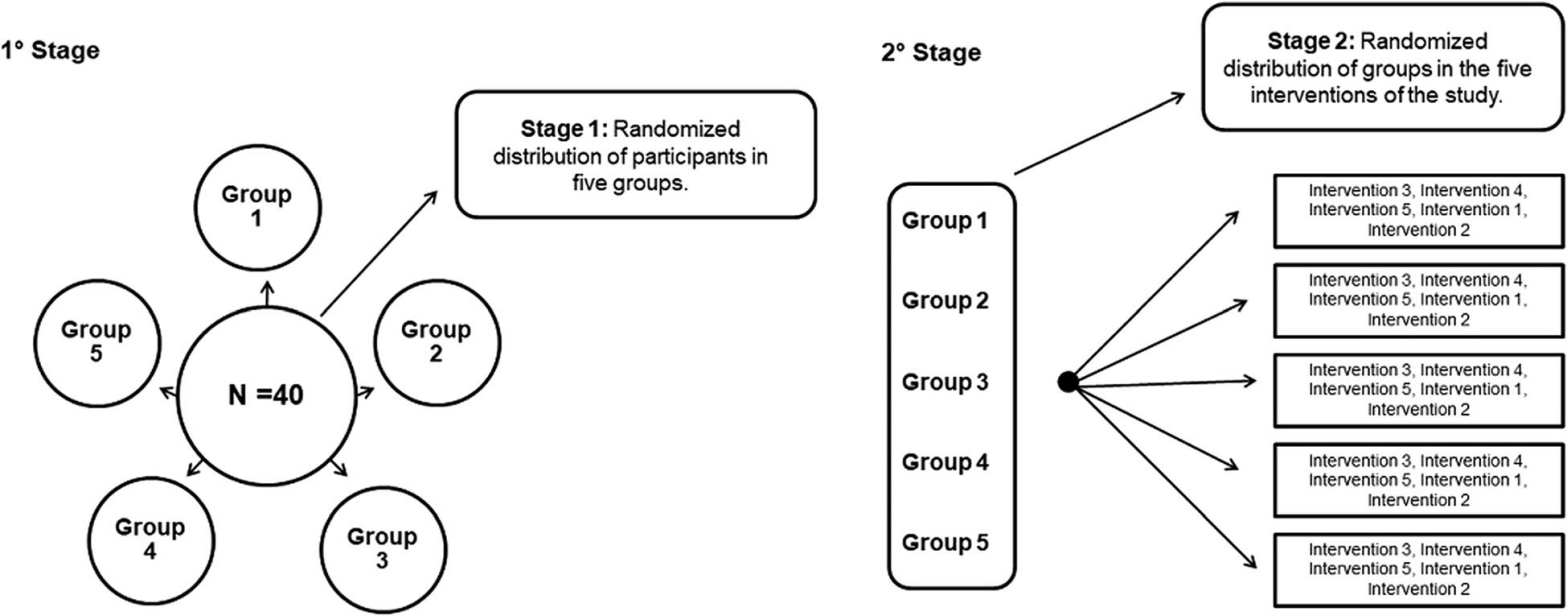
Schematization of the randomization processes

Participants who meet the eligibility criteria and sign the consent form will be randomized, using simple randomization, for logistic reasons into five groups and the groups will be randomized into the different interventions of the study to determine the order of accomplishment. In order to guarantee the hidden allocation of the participants in the groups and the randomization of the study interventions, a researcher not involved in the recruitment will be responsible for this procedure using electronic software (Microsoft Office Excel). This researcher will be instructed not to previously inform the participants or other researchers about the procedures that will be performed in each session. The following figure presents the schematization of the aforementioned randomization processes (Fig. 2).

### Design description

The study will be conducted in five interventions (Intervention 1 (I1); Intervention 2 (I2); Intervention 3 (I3); Intervention 4 (I4); Intervention 5 (I5)), and the groups will be randomized to perform these interventions at several moments, with a 1-week interval between interventions. Data collection will always be performed at the same period of day and in air-conditioned environments.

### Procedures

#### Study interventions

The description of the interventions is as follows:

1. *Intervention 1 (I1)*: the HRV behavior of the participant will be evaluated without exposure to any recovery technique or any type of stress. The follow-up period will be 2 h
2. *Intervention 2 (I2)*: the HRV behavior of the participant will be evaluated before the application of the massage technique. Evaluations will be performed 2 h after massage application
3. *Intervention 3 (I3)*: the HRV behavior of the participant will be evaluated before the implementation of the stress protocol. The autonomic modulation of heart rate will be evaluated 2 h after the end of the stress protocol
4. *Intervention 4 (I4)*: the HRV behavior of the participant will be evaluated before the stress protocol, followed immediately by the massage technique, as well as 2 h after the completion of the massage in order to observe if HRV is anticipated
5. *Intervention 5 (I5)*: the HRV behavior of the participant will be evaluated before the stress protocol, and the massage technique will be applied at the exact moment of ANS recovery, determined by the study of Almeida et al. [11] who used the same stress protocol, and posteriorly there will be follow up for 2 h in order to see if there is potentiation of HRV recovery

The first three interventions of the study (I1, I2, and I3) will be performed to establish the basal behavior of the study variables and/or before the two different stimuli (massage and stress), and for possible explanations and discussion of the study.

In Interventions 4 and 5, analysis of the effects of massage on post-exercise recovery, and the comparison of the effects of massage when performed immediately after stress or 1 h after stress, will be performed.

It is emphasized that at I5 the massage will be applied 60 min after the end of the stress protocol, as this was the exact time identified in the study by Almeida et al. [11] for the recovery of the internal control functions of the body after the same stress protocol as used in the present study.

### Procedure details

#### Stress protocol

Participants will be submitted to a stress protocol consisting of a jumping program and a maximum short-term cycling test [11]. The jumping program consists of 10 sets of 10 jumps with a 1-min interval between sets. During the jump, participants will be instructed to place their hands on their hips and during the jump landing to flex their knees at 90° in order to avoid compensations. After the jump, the participants will have 1 min of rest and then perform the Wingate anaerobic test protocol, which will be performed on a cycle ergometer Biotec 2100 (Cefise, Nova Odessa, Brazil). The test consists of a 5-min warm-up and the test itself. During warm-up the participant will pedal at a speed of 60 to 90 rpm with a fixed load of 1.0 kg, and in the second and fourth minutes the participant will be required to perform a sprint, reaching the maximum possible speed according to individual perception. After the 5-min warm-up the anaerobic test will be performed, consisting of maximum pedaling for 30 s with a stipulated load of 0.075 kp·kg^-1^ of the participant’s body mass. During the sprints and test, verbal stimuli will be provided to stimulate maximum performance.

This stress protocol was chosen due to the study of Almeida et al. [11] which investigated the protocol and concluded that it is effective in triggering derangements in the ANS. Immediately after completion of the protocol, participants will be directed to a stretcher, where they will lie down to perform the massage immediately after stress (I4) or 1 h after stress (I5), or to remain at rest (I1, I2, and I3), and then be followed up for 2 h to capture the RR intervals.

It is worth mentioning that after the stress protocol all participants will be required to drink 180 ml of water in order to standardize hydration and avoid the influence of blood volume on the results of the present clinical trial.

#### Massage protocol

According to a recent systematic review with meta-analysis [19], the effects of massage seem to be better when it is applied for between 5 and 12 min. Thus, the massage in the present protocol will have a total of 12 min duration, being 6 min on the anterior region of the thigh (3 min for each limb) and 6 min on the posterior region of the trunk. The choice of these anatomical sites was due to the effort required by the stress protocol and location of the preganglionic neurons of the ANS, respectively.

For massage on the anterior thigh (quadriceps femoris muscle) the participant will be placed in the dorsal decubitus and the massage initiated on the dominant limb and then the non-dominant limb, and posteriorly, in the ventral decubitus position, massage will be performed on the trunk (paravertebral muscles). The techniques of surface and deep slip will be used, the latter being presented at two intensities, moderate or intense, being gradually increased. The massage will be performed towards the muscle fibers, from distal to proximal and following the lymphatic flow.

The massage will be performed by three physiotherapists specializing in sports physiotherapy, previously trained by a sports physiotherapist with 10 years’ experience in sports massage.

A pilot study will be conducted for the training of the physiotherapists who will apply the massage in a standardized manner regarding the intensity, rhythm, form of application of massage, and application dynamics through data collection. The physiotherapists will be required to present excellent correlations between them in order to be eligible to perform the technique.

The massage will have the rhythm: 1 s of slip and 1 s for return of the hand to the initial position, totaling 2 s of slip (Table 1). The rhythm of the massage will be controlled by an electronic metronome that will only be used by the therapists during performance of the technique.

**Table 1:** Massage protocol for the anterior thigh region and posterior trunk region

Anatomical site	Slip	Pressure	Rhythm	Time (seconds)	Total slips per technique
Anterior thigh	Surface	Light	1 slip every 2 s	60	30
	Deep 1	Moderate	1 slip every 2 s	60	30
	Deep 2	Intense	1 slip every 2 s	60	30
Posterior trunk	Surface	Light	1 slip every 2 s	120	60
	Deep 1	Moderate	1 slip every 2 s	120	60
	Deep 2	Intense	1 slip every 2 s	120	60

Besides standardization of the intensity and rhythm of the massage performed by the physiotherapist, in order not to cause discomfort and possible pressure variations in the application of the technique, participants will be required to report their level of comfort from a scale that considers the technique as “light,” “moderate,” or “intense,” corresponding, respectively, to surface slip, deep 1 slip, and deep 2 slip. Details of the massage protocol can be seen in Table 1.

### Participant timeline

**Fig. 3:**
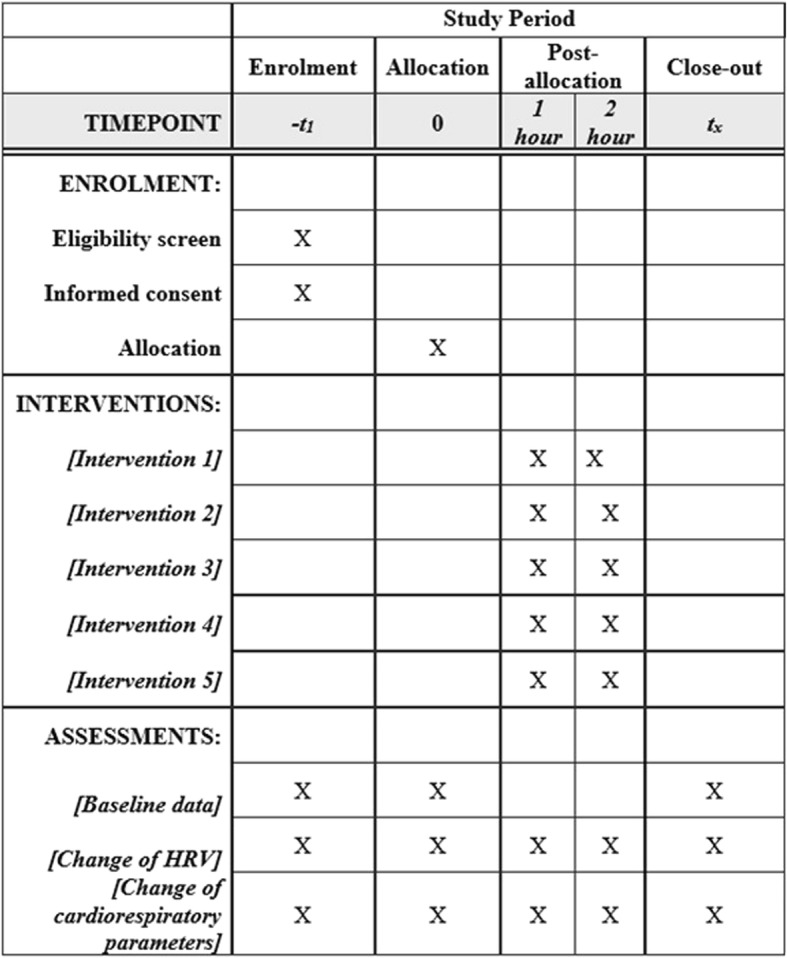
Content for the schedule of enrollment, interventions, and assessments

The time schedule of enrollment, interventions, and assessments is outlined in Fig. 3. Recruitment of study subjects started in April 2018.

### Outcomes

The primary outcome will be the evaluation of autonomic modulation of heart rate by means of HRV. Secondary outcomes are cardiorespiratory parameters (blood pressure, heart rate, respiratory rate, and oxygen saturation) and individual touch perception. Evaluations will be performed by the same independent trained assessors throughout the study.

#### Primary outcome

HRV will be used as the primary outcome as it is an important overall marker of post-exercise recovery [16]. The HRV analysis will be performed from the series of RR intervals captured by the cardiofrequency meter Polar Electro Oy – model V800, and linear methods analyzed in the time domain and frequency domain, and Poincaré plot. All HRV indices will be obtained through the *software* Kubios HRV – version 2.0 [20].

For this analysis, the time series of RR intervals will initially be subjected to digital filtering using the *software* Kubios HRV – version 2.0, supplemented by manual filtering, to eliminate premature ectopic beats and artifacts, and only series with more than 95% of sinus beats will be included in the study [20]. Through visual analysis of the time series, the absence of ectopic artifacts or beats that can interfere in the HRV analysis will be observed.

During the recovery period, the evaluated moments will be: R1 (initial 5 min of rest before performing the interventions), R2 (initial 5 min of recovery), R3 (fifth to 10th minutes of recovery), R4 (10th to 15th minutes of recovery), R5 (20th to 25th minutes of recovery), R6 (30th to 35th minutes of recovery), R7 (40th to 45th minutes of recovery), R8 (50th to 55th minutes of recovery), R9 (60th to 65th minutes of recovery), R10 (70th to 75th minutes of recovery), R11 (90th to 95th minutes of recovery), and R12 (115th to 120th minutes of recovery). It is worth mentioning that 256 consecutive RR intervals will be obtained at these moments [21].

HRV indices in the time domain (rMSSD and SDNN), frequency domain (low-frequency spectral component (LF) and high-frequency spectral component (HF)), and the Poincaré plot (SD1 and SD2) will be evaluated [22].

In the time domain, the rMSSD Index corresponds to the square root of the square mean of the differences between the adjacent normal RR intervals in a time interval expressed in milliseconds and evaluates the behavior of the parasympathetic autonomic modulation [22], while the SDNN Index represents the standard deviation of all normal RR intervals recorded in a time interval, expressed in milliseconds, and is considered a measure of global variability, that is, it evaluates both sympathetic and parasympathetic modulation [22].

In the frequency domain the low-frequency spectral component (LF – frequency between 0.04 and 0.15 Hz) is considered an index that evaluates sympathetic and parasympathetic behavior [22, 23], while the high-frequency spectral component (HF – frequency between 0.15 and 0.4 Hz) is a marker of vagal modulation [22, 23]. The spectral analysis will be calculated from a tachograph using the fast Fourier transform algorithm.

The Poincaré plot is a map of points in Cartesian coordinates constructed from the values of the RR intervals obtained [22]. For quantitative analysis of the plot an ellipse will be adjusted to the points of the graph where the indices SD1 and SD2 are obtained. SD1 is an instantaneous index of beat-to-beat variability and represents the parasympathetic modulation [24], while the SD2 Index represents the HRV in long-term records, and reflects the overall variability [24].

#### Secondary outcomes

The study will include five measures of secondary outcomes: individual touch perception, which will be collected at baseline of the first randomized intervention, and blood pressure, heart rate, respiratory rate, and oxygen saturation, which will always be collected at baseline and during the 2 h of recovery at minutes 1, 3, 5, 7, 10, 20, 30, 40, 50, 60, 70, 80, 90, 100, 110, and 120. In addition, in I5 these variables will be collected 1 h prior to the application of the massage at minutes 1, 3, 5, 7, 10, 20, 30, 40, 50, and 60.

- ► *Blood Pressure*
  - ○ Blood pressure will be verified by an indirect method using a stethoscope (Littmann, Saint Paul, MN, USA) and an aneroid sphygmomanometer (Welch Allyn – Tycos, Skaneateles Falls, NY, USA) fixed on the left arm of the volunteers. To avoid errors in determining blood pressure, these measurements will be performed by a single evaluator. The measurements will be performed according to the criteria established by the VII Brazilian Hypertension Guidelines [25]
- ► *Heart rate*
  - ○ To verify the heart rate, a cardio frequency meter (Polar Electro Oy, Kempele, Finland – model V800) will be used [22]
- ► *Respiratory rate*
  - ○ Respiratory rate measurements will be performed by counting the respiratory incursions for 1 min without the participant being aware of the process, so that the usual breathing characteristics are not modified [26]
- ► *Oxygen saturation*
  - ○ Oxygen saturation will be verified by means of a pulse oximeter (Mindray PM-50 Pulse Oximeter, China). The pulse oximeter is a device that provides blood saturation readings by evaluating the absorption behavior of oxyhemoglobin and deoxyhemoglobin in relation to red and infrared light lengths [27]
- ► *Questionnaire on individual touch perception*
  - ○ The *Touch Avoidance Questionnaire* [28] is a questionnaire composed of 37 questions that evaluate the experience of the touch received by another person. One question addresses how invasive the touch is from the unknown person and the other question addresses the touch experience by a professional whose profession requires the same touches, such as masseurs. The questionnaire contains 37 questions, and the questions offer the alternatives: “true” and “false” for scoring the touch experience; however, we will only use two adapted questions and the answers will be given on a linear scale equivalent to 10 cm, where 0 is without any discomfort and 10 is extremely uncomfortable

### Sample size

As described above the primary outcome will be the HRV. The sample size calculation was carried out based on the study of Arroyo-Morales et al. [15] in which the SDNN Index of the HRV was selected, since it is an index that expresses the behavior of the ANS in a global way. To establish the sample size, a mean effect of 26.9 and standard deviation of 38.8 were chosen. The level of significance for the sample size is 5%, the power test is 80%, two-tailed test, and 10% of the total value of *n* is added to statistically supply the analyses in the case of withdrawals during data collection. The value obtained for the sample size was 33 participants, adding the 10% gave a sample of 36 volunteers. However, there are other indices besides the SDNN Index within the HRV, and this situation generates an environment where the sample size could be underestimated. In the same sense, due to the new proposal presented by the study, there are no trials in the literature that have the same proposal dynamics, and thus, for safety we will add four volunteers to ensure the statistical analysis of all HRV indices, thus totaling a sample of 40 participants.

### Statistical analysis

For analysis of the data of the population profile, the descriptive statistical method will be used and the results presented as values of means, standard deviations, median, minimum and maximum numbers, and confidence interval. The normality of the data will be evaluated through the Shapiro-Wilk test.

The comparisons of cardiorespiratory parameters and HRV indices between interventions (I1, I2, I3, I4, and I5) and moments (rest (R1) vs recovery moments (R2–R12)), the data are analyzed from the prism of the covariance analysis (ANCOVA), which will be adjusted for the baseline value of the evaluated variable and generated estimated means after such adjustment (extracting the variance explained by the confounding variables). The Levene test will be used to prove the homogeneity of the variances at the created moments, and the Bonferroni post hoc test will be used for comparison. To guarantee homogeneity of data the variable can will be transformed into logarithm.

For analysis of the moments (rest vs recovery moments), repeated measures ANOVA with the Tukey post test for parametric distribution or Friedman test with Dunnett’s post test for non-parametric distribution will be used and the analysis of the different groups will be performed by means of one-way ANOVA with the Tukey post test or the Kruskal-Wallis test with Dunnett’s post test.

The level of significance will be *p* < 0.05 for all tests. The SPSS statistical program (version 13.0) (SPSS Inc., Chicago, IL, USA) will be used for the analyses. Data integrity will be monitored by regularly scrutinizing data files for omissions and errors. Participants will be given an anonymous study ID to protect confidentiality, and only study investigators will have access to the final trial data set.

## Discussion

### Potential impact and significance of the study

This massage protocol was created from recent findings in the literature and its standardization was performed according to the union of the best results found. The implementation and use of this standardized protocol will provide important and reliable information regarding the use of massage in post-exercise recovery, with the identification of its effects on the ANS and the best moment and form of massage application, representing an important advance in the sports field and in clinical practice.

### Strengths and limitations of this study

A strong point of this study is that the protocol created was based on the latest in the literature on the theme. Another strong point of the study is the comparison of the effects of massage when applied at different moments on post-exercise recovery. In addition, its design of a randomized clinical trial of the cross-over type, the easy reproducibility of the protocol from the description of the same, the fact of the massage being performed by three physiotherapists specialized in sports physiotherapy also enhances the study. However, one limitation of this study is the impossibility of blinding the physiotherapists and participants regarding the application of the technique, and although standardized there is no control of the pressure exerted during the massage, only a subjective control through the level of comfort of the volunteer with the specific scale application.

### Contribution and clinical applicability

With the accomplishment of this study we will verify which HRV indices demonstrate a significant influence of the massage in post-exercise recovery, and which moment of application presents better recovery after exercise. The data obtained in the present study will provide subsidies for the best management of application of the technique in sports clinical practice, considering periods of training and, mainly, of competitions.

## Trial status

Number Protocol: NCT03094676

Patient recruitment is currently underway.

Study start date: 9 April 2018

Primary completion date: 14 May 2018

Study Completion date: 30 July 2018

## Additional file

Additional file 1: Recommendations for Interventional Trials (SPIRIT). (DOC 120 kb)

## Abbreviations

ANS: Autonomic nervous system
BMI: Body Mass Index
FCT/UNESP: Universidade Estadual Paulista
HF: High-frequency spectral component
HRV: Heart rate variability
I1: Intervention 1
I2: Intervention 2
I3: Intervention 3
I4: Intervention 4
I5: Intervention 5
IPAQ: International Physical Activity Questionnaire
LF: Low-frequency spectral component
rMSSD: Index corresponds to the square root of the square mean of the differences between the adjacent normal RR intervals in a time interval, expressed in milliseconds
SD1: Instantaneous index of beat-to-beat variability
SD2: Index represents the HRV in long-term records
SDNN: Index represents the standard deviation of all normal RR intervals recorded in a time interval, expressed in milliseconds
